# An Investigation of Factors Affecting Elementary School Students' BMI Values Based on the System Dynamics Modeling

**DOI:** 10.1155/2014/575424

**Published:** 2014-02-20

**Authors:** Tian-Syung Lan, Kai-Ling Chen, Pin-Chang Chen, Chao-Tai Ku, Pei-Hsuan Chiu, Meng-Hsiang Wang

**Affiliations:** ^1^Department of Information Management, Yu Da University of Science and Technology, Miaoli 36143, Taiwan; ^2^Department of Leisure Recreation and Travel Management, Toko University, Chiayi 61363, Taiwan; ^3^Department of Leisure Management, Yu Da University of Science and Technology, Miaoli 36143, Taiwan

## Abstract

This study used system dynamics method to investigate the factors affecting elementary school students' BMI values. The construction of the dynamic model is divided into the qualitative causal loop and the quantitative system dynamics modeling. According to the system dynamics modeling, this study consisted of research on the four dimensions: student's personal life style, diet-relevant parenting behaviors, advocacy and implementation of school nutrition education, and students' peer interaction. The results of this study showed that students with more adequate health concepts usually have better eating behaviors and consequently have less chance of becoming obese. In addition, this study also verified that educational attainment and socioeconomic status of parents have a positive correlation with students' amounts of physical activity, and nutrition education has a prominent influence on changing students' high-calorie diets.

## 1. Introduction

As indicated by statistics of elementary school students' health checks each year, being overweight or underweight has a certain degree of adverse effects on one's body. This study proposes to conduct an investigation on the health issues of elementary school students and detect factors that affect these students' body mass index (BMI) values [1]. Many students prefer high-calorie diets, which not only cause excessive calorie intake but also increase burdens to one's body. Obesity has the tendency to incur a variety of chronic diseases and has a significant impact on students' physical health [2]. This study aims to encourage schools to advocate and implement nutrition education; thus, students can learn proper eating attitudes and establish the healthy concepts of a balanced healthy diet and further reduce the incidence of chronic diseases [3].

This study mainly consists of research on the four dimensions: student's personal life style, diet-relevant parenting behaviors, advocacy and implementation of school nutrition education, and students' peer interaction [4]. An exploration of the contributing factors to students' BMI values is conducted from diverse perspectives [5]. In addition, a number of themes that merely utilized the conventional approach of qualitative and static discussion and analysis are integrated in this study to have dynamic simulation and discussion, and the discourse generated through the discussion is analyzed from different angles. Primarily assuming a dynamic perspective and using the Vensim software in model construction and simulation, this study further analyzes and discusses the complex issues in real life with reference to the numerical changes and the liner trends in all models, and clarifies the causal relationship between each variable and students' BMI values [6].

## 2. Research Method

In order to have an exact understanding of the dynamic relationship between each influencing factor and students' BMI values, this study is conducted by utilizing the Vensim software. The operation of system dynamics and simulation through computer programing enables a better grasp of various routes of feedbacks and characteristics of time delay in the process of changes in the system. The Vensim software, which was developed from the Ventana Systems, Inc., is able to provide researchers a simple and flexible method to construct relevant model structures such as a causal and feedback loops and a flow chart. This software can also be employed to construct a dynamic model by using equations to write the quantitative correlation between each variable and parameter into the model [7]. The Vensim software is a graphical interface software that can simulate and analyze a system dynamics model, implying that as long as the graphical arrow marks are connected with the marks of various kinds of variables, and a suitable equation is applied to write the relationship between each variable into the model, the causal relationship between each variable is recorded and completed [8]. Through the establishment of a dynamic model, users can understand the causal relationship and feedback loop of each variable as well as understand the input and output relationship between each variable through the special features of the program. Further, users can have a better command and clear view of the structure of the entire model, and inventor of the model can easily modify the content of the model.

The Vensim software is used to construct the dynamic model as follows [9–11].

1. Confirm the theme of exploration and the desired objectives.
2. Observe and describe the investigated issues, collect relevant data and studies as supporting evidence, locate a variety of variables that are related to these issues, and further interpret the correlation between each variable.
3. Convert the qualitative variables in the system into numerical values for easier representation, as well as use mathematic algorithms to define and interpret the casual and feedback relationship of variables in the system.
4. Use computer simulation software to simulate the setting of each variable in the system.
5. Verify and interpret the simulation results to confirm whether the results conform to the expected objectives.
6. Modifications are required in the event where the simulation results fail to conform to the actual system or hypothesis until the results can be presented clearly to interpret issues and objectives.
7. Repeat the experiment, in which the above steps continue to undergo tests and modifications until the results can be clearly represented. Once the issues can be interpreted, the objectives can be achieved.

**Figure 1:**
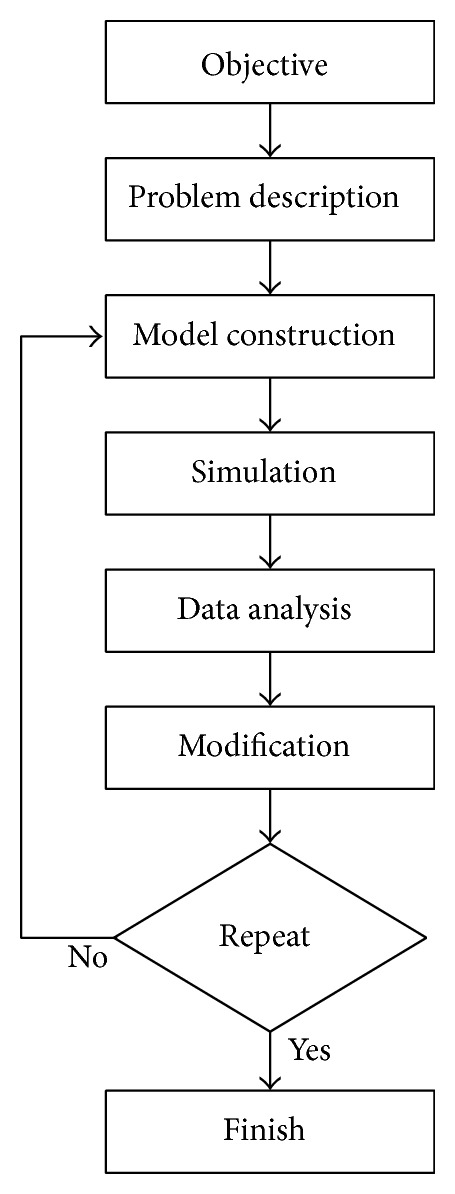
Research process flowchart.

The research process of this study is shown in Figure 1.

## 3. Model Construction

The construction of the dynamic model is divided into the qualitative causal loop and the quantitative system dynamics modeling as follows.

(1) The qualitative causal loop: in this study, a causal loop that consists of the contributing factors to students' BMI values is established, which encompasses diet-associated parenting behaviors, students' perception of self-body image, the effectiveness of the implementation of school nutrition education, and students' experience of being ridiculed due to their body shapes.

**Figure 2:**
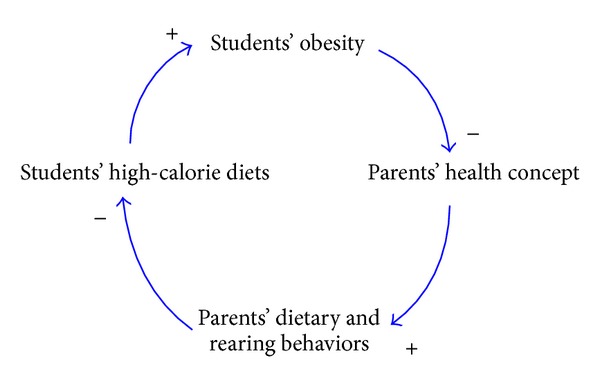
The causal loop diagram consisting of diet-associated parenting behaviors and students' BMI values.

Diet-associated parenting behaviors have a significant influence on students' eating habits. As students who come from a higher socioeconomic family background and have parents with better concepts of health usually have a more balanced diet than students from a lower socioeconomic background. Therefore, students with more abundant nutrition-related knowledge and healthier food preference also have better eating behaviors. In this study, factors pertaining to diet-associated parenting behaviors and students' BMI values are turned into a causal loop diagram, as shown in Figure 2.

**Figure 3:**
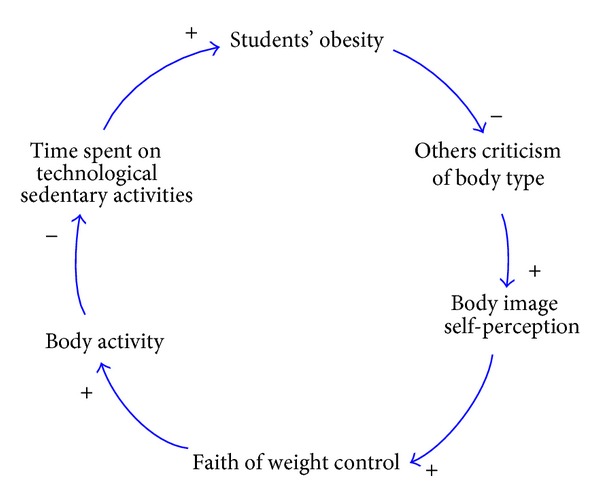
The causal loop diagram consisting of students' perception of self-body image and students' BMI values.

Students with a higher body satisfaction and a more positive body image tend to pay more attention to their own appearance and demonstrate healthier behaviors in having balanced and low-calorie diets in order to keep their good body shapes. In addition, there is also a significant correlation between students' satisfaction with their body parts and the amount of their physical activity, which indicates a positive correlation between students' perception of self-body image and their BMI values. In this study, students' self-body image perception and students BMI were turned into a casual loop diagram, as shown in Figure 3.

**Figure 4:**
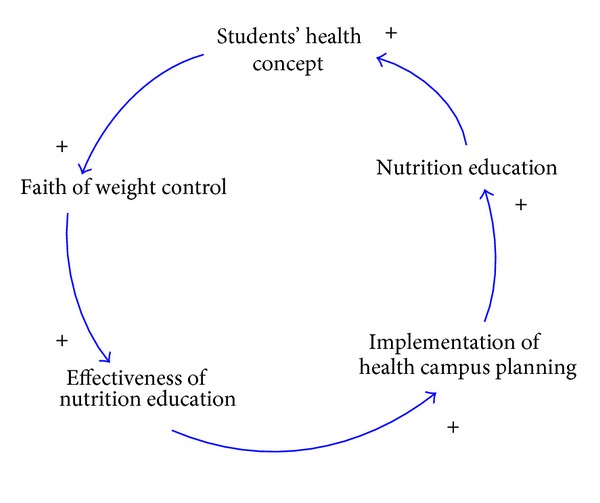
The causal loop diagram consisting of the effectiveness of the implementation of school nutrition education and students' concepts of health.

The intervention and mentoring of school nutrition education has a certain degree of effect on improving students' nutrition knowledge and dietary behaviors, implying that the implementation of school nutrition education can significantly enhance students' dietary behaviors and concepts of health. In this study, a causal loop diagram consisting of factors pertaining to the effectiveness of the implementation of school nutrition education and students' concepts of health is shown in Figure 4.

**Figure 5:**
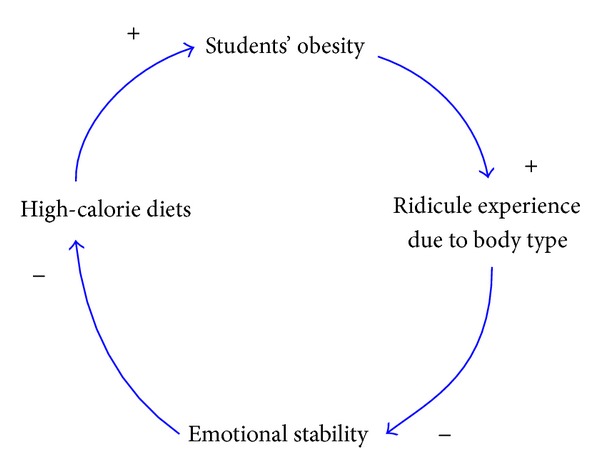
The causal loop diagram consisting of students' experience of being ridiculed due to their body shapes and students' high-calorie diets.

In addition to a better chance of having eating disorders than students with normal body weights, students with an obese body shape are also more vulnerable to the influence of external aesthetic value and their own body image. On the contrary, students with a more positive body image are usually more aware of better low-calorie diet tips. Besides, there is a negative correlation between students' evaluation of their own appearance and their BMI values; that is, students with a more negative evaluation of their own appearance are more likely to have high-calorie diets. A causal loop diagram consisting of factors in relation to students' experience of being ridiculed due to their body shapes and students' high-calorie diets is show in Figure 5.

**Figure 6:**
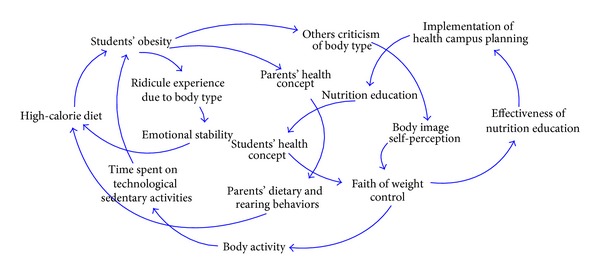
The causal loop diagram consisting of students' BMI values.

In summary, an integrated causal loop diagram of students' BMI values, as shown in Figure 6, is formed by incorporating the above four subcasual loop diagrams.

(2) Quantitative system dynamics modeling: the system dynamics models, which are constructed based on the above casual loop diagrams, are shown in the following paragraphs.

**Figure 7:**
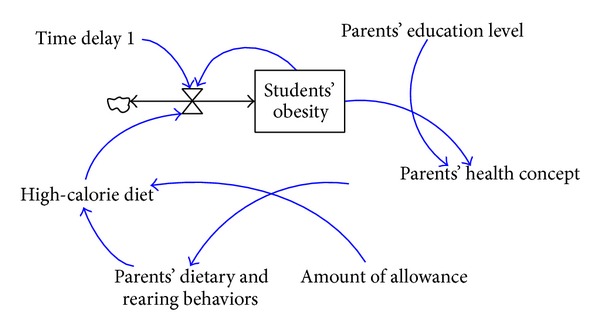
The system dynamics model consisting of diet-associated parenting behaviors and students' BMI values.

A higher educational attainment or socioeconomic status of parents indicates more accurate nutrition-related knowledge and better concepts of health. Parents' accurate concepts of nutrition would affect parental standards on children's diets and further parental behaviors of encouraging students to keep a normal body weight and develop healthy dietary behaviors. A system dynamics model that illustrates the relationship between diet-associated parenting behaviors and students' BMI values is shown in Figure 7.

**Figure 8:**
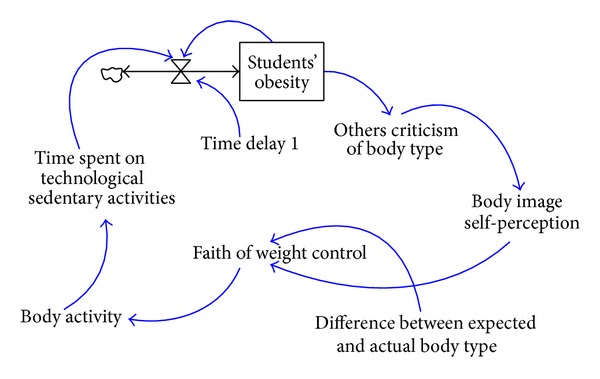
The system dynamics model consisting of students' perception of self-body image and students' BMI values.

The lower BMI value a student has, the higher body evaluation the student usually receives from parents or peers, which results in his/her more positive self-body image. Students who have a more positive body image, attach more importance to appearance, and pay more attention to one's own body weight also enjoy more balanced diets and a relatively higher incidence of having highly-disciplined eating behaviors. A system dynamics model, as shown in Figure 8, is formed to illustrate the relationship between students' body image and students' BMI values.

**Figure 9:**
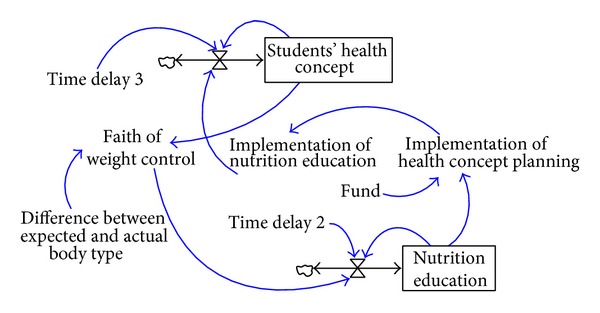
The system dynamics model consisting of the effectiveness of the implementation of school nutrition education and students' health concepts.

A better performance of a healthy campus promotional program indicates a more successful advocacy and implementation of school nutrition education. As students possess more adequate and accurate nutrition-related knowledge, they would have better concepts of health. Through elaboration in related school courses, students can learn the importance of weight management and further adopt healthy eating behaviors. Therefore, students' stronger belief in weight management indicates a higher effectiveness of the implemented school nutrition education. In this study, the correlation between the effectiveness of the implantation of school nutrition education and students' concepts of health is shown in the system dynamics model in Figure 9.

**Figure 10:**
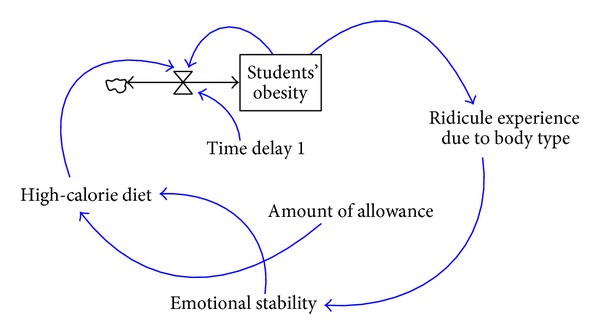
The system dynamics model consisting of students' experience of being ridiculed for their body shapes and students' high-calorie diets.

Students with lower BMI values usually receive a higher parental and peer evaluation, have a more positive body image, and have less experience of being ridiculed. A higher degree of parental and peer support and less experience of being ridiculed make the emotional stability of students with lower BMI values relatively higher than the emotional stability of obese students, and are less likely to have high-calorie dietary behaviors due to emotion-related reasons. In this study, the correlation between students' experience of being ridiculed and students' high-calorie diets is shown in a system dynamics model in Figure 10.

**Figure 11:**
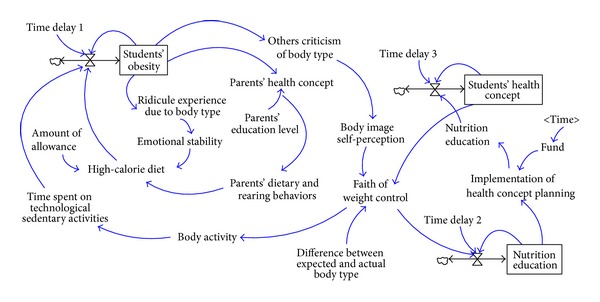
The system dynamics model consisting of students' BMI values.

As an integration of the above results, the four dynamic models are integrated into a more complex system dynamics model consisting of students' BMI values, as shown in Figure 11.

## 4. Simulation Results

In this study, simulation tests in accordance with the above system dynamics model are conducted to probe into the correlation between factors such as students' concept of health, obesity, educational attainment and socioeconomic status of parents, amounts of students' physical activity, students' high-calorie diets, and the effectiveness of the implementation of school nutrition education.

**Figure 12:**
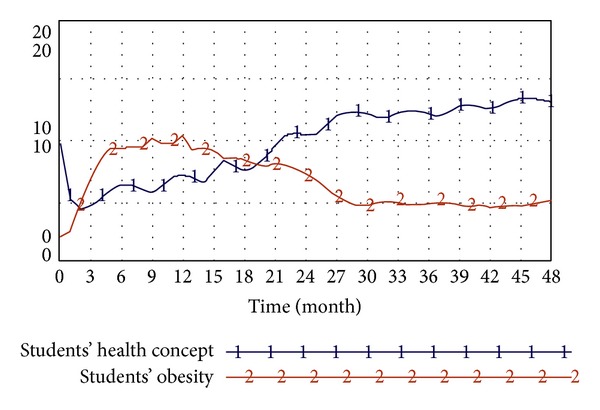
Analysis of the influence of students' health concepts on students' degree of obesity.

**Figure 13:**
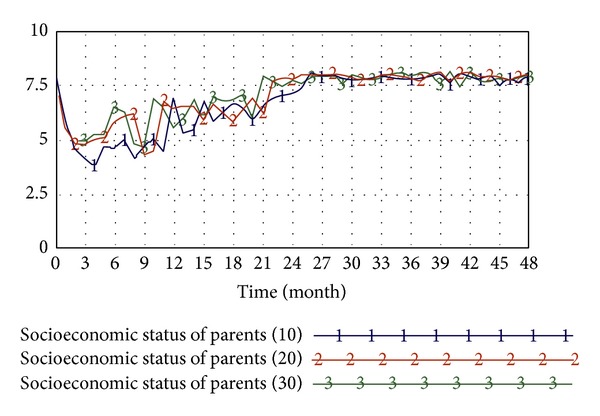
Analysis of the influence of educational attainment and socioeconomic status of parents on the physical activities of students.

**Figure 14:**
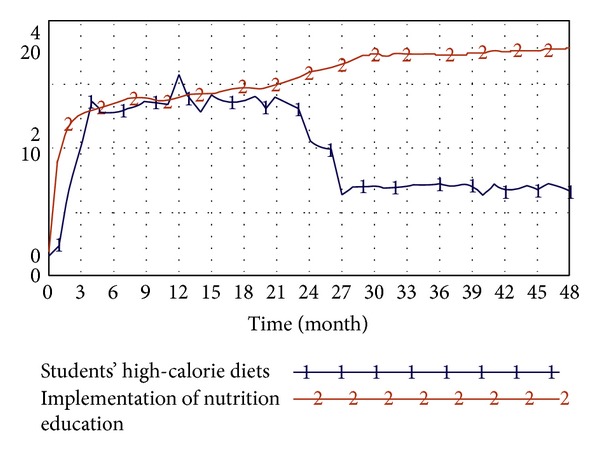
Analysis of the influence of implemented school nutrition education and students' high-calorie diets.

(1)  The influence of students' concepts of health on obesity: the system simulation primarily investigates the correlation between students' concepts of health and obesity. As revealed by the simulation results, students with better concepts of health have a lower degree of obesity. As shown in Figure 12, despite the slightly different liner changes in the first half part of the diagram, the liner changes in the second half part are in conformability, indicating a negative correlation between students' concepts of health and obesity.
(2)  The influence of the educational attainment and socioeconomic status of parents on the amount of physical activity of students: the system simulation primarily investigated the correlation between the educational attainment and socioeconomic status of parents and the amount of physical activity of students. The result, as shown in Figure 13, shows a positive correlation between the educational attainment and socioeconomic status of parents and the amount of students' physical activity.
(3)  The influence of the implementation of school nutrition education on students' high-calorie diets: the system simulation primarily investigates the correlation between students' high-calorie diets and the effectiveness of the implemented school nutrition education. As indicated by the result, there is a positive correlation between the two variables. As shown in Figure 14, such correlation between school nutrition education and younger students is even more prominent as younger students, whose diet patterns haven't been completely shaped, are more susceptible to educational influence.

## 5. Conclusion

This study verified that students with better concepts of health have a lower level of obesity, and there is a negative correlation between health concepts and obesity. In addition, younger students might have weaker self-discipline over ones' own behaviors and are therefore more vulnerable to the influence of peers. To satisfy their desire for tasty food, younger students sometimes eat unhealthy snacks or drink unhealthy drinks. Students who are not disciplined at a young age are not only prone to develop a poor eating habit but also have an increased risk of obesity. To cope with this problem, parents should prepare fruits or crackers as children's snacks and instruct them the proper nutrition concepts to avoid their excessive intake of calories. On the other hand, schools can gradually improve students' diet tips and nutrition-related knowledge through the advocacy of nutrition education. Students with more adequate concepts of health usually have better eating behaviors and attitudes and consequently have less chance of becoming obese.

Secondly, this study verified that the educational attainment and socioeconomic status of parents have a positive correlation with students' amounts of physical activity. Parents with a high socioeconomic background usually have a better concept of health. They are willing to pay more attention to children's mental and physical development as well as spend more time and money to give children more learning opportunities such as organizing children to participate in school club activities or to learn additional skills. Some parents even seize the after-school time to accompany children to do exercise for the purposes of fitness and parent-child interaction. Therefore, parents having a high educational attainment and socioeconomic status usually imply a higher amount of physical activity of their children.

Finally, this study verified that the implementation of school nutrition education has a certain effect on curbing the frequency of students' intake of high-calorie diets. There is a negative correlation between the two actions. To create a healthy learning environment so students can study at peace, schools have usually devoted unreserved efforts into the advocacy of nutrition education. In addition to the routine arrangement of relevant courses, random education is also conducted during lunch time, so students can develop proper knowledge of nutrition. In addition, full-time school dietitians can design a menu based on the nutritional needs of students to guard the health of students. As for school lunch, it is served by the school's central kitchen, which changes dishes regularly and accordingly after consultation with school teachers and students in the hope that students can enjoy the nutritious meals. Teachers also remind students to buy fewer drinks and have deep-fried foods as little as possible so students can develop good eating habits since childhood. All in all, this study has proved that nutrition education has a prominent influence on changing students' high-calorie diets.
